# Endothelial Leakage–Erythrocyte Stress Index for the prediction of pre-eclampsia in the first trimester: A preliminary, hypothesis-generating retrospective case-control study

**DOI:** 10.1097/MD.0000000000050525

**Published:** 2026-09-04

**Authors:** Funda Demirel, Berrin Oztas

**Affiliations:** a Department of Gynecology and Obstetrics, Kocaeli City Hospital, Kocaeli, Turkey; b Department of Biochemistry, Faculty of Medicine, Kocaeli University, Kocaeli, Turkey.

**Keywords:** ELES index, endothelial dysfunction, first trimester, oxidative stress, pre-eclampsia

## Abstract

Pre-eclampsia is a major cause of maternal and perinatal morbidity, and there remains a need for reliable and easily accessible biomarkers for early prediction. The Endothelial Leakage–Erythrocyte Stress (ELES) index is a novel composite biomarker derived from routine first-trimester red cell distribution width (RDW), aspartate aminotransferase (AST), and serum albumin, reflecting endothelial leakage and erythrocyte stress. This retrospective case-control study evaluated the predictive value of the ELES index for pre-eclampsia in singleton pregnancies undergoing routine laboratory assessment at 11–13 + 6 weeks who delivered at the same hospital. First-trimester ELES index values were significantly higher in women who later developed pre-eclampsia than in controls (6.36 ± 2.59 vs 4.75 ± 1.38, *P* < .001), with an AUC of 0.75 (95% CI: 0.65–0.85); at a cutoff of ≥5, sensitivity and specificity were 76.3% and 65.0%, respectively. After adjustment for maternal age and obstetric characteristics, ELES ≥ 5 remained independently associated with pre-eclampsia (OR 5.971, 95% CI: 2.427–14.688, *P* = .001); higher AST and lower albumin were also independently associated. Albumin alone yielded the highest AUC (0.775, 95% CI: 0.689–0.847), and the ELES index was not statistically superior to albumin alone (DeLong *P* = .698). Because first-trimester albumin is not part of routine national antenatal screening, only women in whom this measurement had already been requested could be analyzed, and BMI, aspirin prophylaxis, gestational age at diagnosis, and disease severity were not retrievable. These findings should therefore be regarded as preliminary and hypothesis-generating, requiring prospective validation in a broader obstetric population before clinical use.

## 1. Introduction

Pre-eclampsia is one of the leading causes of maternal and perinatal morbidity and mortality, which emerges after the 20th week of pregnancy, and is characterized by hypertension and often, proteinuria.^[1]^ Recent evidence has shown that abnormal placentation, widespread endothelial dysfunction, and the systemic inflammatory response play a fundamental role in the pathogenesis of pre-eclampsia.^[2,3]^

In a normal pregnancy, low-resistance uteroplacental circulation is provided by trophoblastic invasion of the spiral arteries, whereas in pre-eclampsia this process remains insufficient and placental hypoxia develops. Anti-angiogenic factors released from hypoxic placenta, especially an increase in soluble fms-like tyrosine kinase-1 (sFlt-1), and a decrease in placental growth factor (PlGF) lead to widespread endothelial dysfunction in the maternal circulation.^[4,5]^ A deterioration in endothelial cell integrity causes a weakening of intercellular connections and increased vascular permeability leading to endothelial leakage.^[6]^ Through intravascular protein loss, this condition results in decreased levels of serum albumin. The resultant hypoalbuminemia is not only a hepatic synthesis disorder but is also evaluated as an important biochemical marker reflecting disruption of systemic endothelial integrity.^[7]^

Another important mechanism accompanying endothelial dysfunction is erythrocyte deformation triggered by microvascular damage and oxidative stress. Increased vascular resistance and oxidative stress cause increased erythrocyte heterogeneity by disrupting erythrocyte membrane integrity. This can be indirectly evaluated by an increase in red cell distribution width (RDW) on the routine haemogram.^[8,9]^ Although a relationship has been shown between increased RDW and pre-eclampsia, the vast majority of studies in the literature have focused on the later weeks of pregnancy when the disease has become clinically more evident.^[10]^ Aspartate aminotransferase (AST) is accepted as one of the early biochemical markers of cellular damage due to hepatic sinusoidal endothelial damage and microvascular ischemia in pre-eclampsia and may start to rise in the subclinical period.^[11]^ Therefore, evaluation of AST is important not only as a parameter reflecting hepatic damage but also systemic endothelial dysfunction.

Various haematological indexes reflecting inflammation and endothelial dysfunction in the pathogenesis of pre-eclampsia have been investigated in recent years. Inflammation-based markers such as the neutrophil-lymphocyte ratio (NLR), platelet-lymphocyte ratio (PLR), and systemic immune-inflammation index (SII) have been associated with pre-eclampsia; however, these markers mainly reflect systemic inflammation and may inadequately represent endothelial dysfunction and microvascular injury, particularly in early pregnancy.^[10,12]^ The Endothelial Activation and Stress Index (EASIX) is one of the composite indexes targeting endothelial dysfunction, which was developed to reflect microvascular damage and has prognostic value in conditions such as transplantation and critical diseases.^[13]^ However, in predicting pre-eclampsia in pregnancy, especially in the first trimester, the efficacy of EASIX is limited, and it does not directly include erythrocyte stress.

The objective of this study was to investigate whether routinely available first-trimester laboratory parameters, obtained before the clinical onset of pre-eclampsia, could identify women at increased risk for subsequent disease development. To this end, we evaluated the predictive value of a novel composite index, the Endothelial Leakage–Erythrocyte Stress (ELES) Index, derived from RDW, AST, and albumin levels measured in the first trimester.

## 2. Materials and methods

### 2.1. Study design and population

This retrospective case-control study was conducted at Kocaeli City Hospital, a tertiary-level training and research hospital in Turkey. The study was performed between December 2025 and April 2026. The source population comprised all women who gave birth at Kocaeli City Hospital during the 3-year period preceding the study. The sampling frame was constructed by cross-referencing the labor-ward delivery register with the laboratory information system. Eligibility required a singleton pregnancy, a first-trimester (11–13 + 6 weeks) laboratory panel performed in our own central laboratory that contained RDW, AST, and albumin, and delivery at the same institution. Every woman who satisfied these criteria and had none of the exclusion criteria during the study period was enrolled; no random selection, matching, quota sampling, or stratification was applied, and no eligible case was omitted. Cases were defined by an International Society for the Study of Hypertension in Pregnancy (ISSHP)-based diagnosis of pre-eclampsia recorded in the delivery file, and controls were the consecutively identified normotensive women who delivered during the same calendar period, so that cases and controls arose from the same source population and were exposed to identical laboratory methods, instruments, and secular clinical practice. Approval for the study was granted by the Local Ethics Committee (2026-60).

### 2.2. Study inclusion and exclusion criteria

The study included women with singleton pregnancies who underwent routine hematological and biochemical evaluation at 11–13 + 6 weeks of gestation and delivered at the same institution. Cases consisted of women who subsequently developed pre-eclampsia during pregnancy. Controls consisted of women who remained normotensive throughout pregnancy and delivered healthy neonates during the same study period.

Cases were excluded if they had multiple pregnancy, chronic hypertension, diabetes mellitus, liver disease, renal disease, hematological disease, active infection, or incomplete clinical records.

### 2.3. Study size

No formal sample size calculation was performed because of the retrospective design of the study. All eligible women meeting the inclusion criteria during the study period were included.

### 2.4. Data collection

Demographic data (age, gravida, parity), obstetric outcomes (gestational age at birth, birthweight, manner of birth), and laboratory parameters were obtained retrospectively from the hospital records system.

The laboratory parameters recorded in the first trimester included hemogram parameters (RDW, neutrophils, lymphocytes, platelets, mean platelet volume [MPV]) examined with a Sysmex XN-1000 analyzer (Sysmex Inc, Kobe, Japan) and biochemical parameters (AST, alanine aminotransferase [ALT], albumin) examined with a Cobas c701 analyzer (Roche Diagnostics, Penzberg, Germany).

Internal quality control was performed daily at 2 concentration levels for the biochemistry analytes (AST, albumin) and 3 concentration levels for the hematology analyte (RDW), with Westgard multi-rule evaluation applied; any results falling outside acceptable control limits were rejected and reanalyzed. Intra-assay and inter-assay coefficients of variation were routinely recorded for all 3 analytes. In addition, continuous participation in external quality assessment programs was maintained for all 3 parameters, and the analytical methodology and metrological traceability of each assay were provided by the manufacturer.

To minimize information bias, all laboratory measurements were obtained from standardized hospital records and analyzed using the same laboratory platforms throughout the study period. Uniform ISSHP diagnostic criteria were applied to all participants for the diagnosis of pre-eclampsia. Consecutive eligible women meeting the predefined inclusion and exclusion criteria were included to reduce selection bias.

### 2.5. Pre-eclampsia diagnosis

The diagnosis of pre-eclampsia was established according to the criteria of the International Society for the Study of Hypertension in Pregnancy (ISSHP).^[14]^ The presence of organ dysfunction or proteinuria (≥300 mg/24 h) accompanying new-onset hypertension (≥140/90 mm Hg) developing after the 20th week of pregnancy was accepted as pre-eclampsia.

### 2.6. Calculation of the indexes

The ELES index was calculated using the RDW, AST, and albumin parameters to reflect endothelial leakage and erythrocyte stress together. The ELES index was calculated using the following formula: (RDW × AST)/serum albumin. RDW was recorded as a percentage (%), AST and ALT as units per liter (U/L), and serum albumin as grams per liter (g/L), according to the standard laboratory reporting system of our institution. In addition, to evaluate systemic inflammation, the neutrophil/lymphocyte ratio (NLR) and the thrombocyte/lymphocyte ratio (PLR) were calculated.

The ELES index was structurally inspired by the EASIX, which is a composite biomarker model reflecting endothelial dysfunction and microvascular injury.^[13]^ The formulation of the ELES index was based on pathophysiological reasoning rather than empirical mathematical optimization. RDW and AST were positioned in the numerator because both parameters are expected to increase in association with oxidative stress, cellular injury, and microvascular dysfunction in pre-eclampsia.^[8,9,11]^ The multiplicative interaction between RDW and AST was intended to reflect the combined hematological and hepatic endothelial injury burden. In contrast, albumin was positioned in the denominator because lower albumin levels are considered to reflect endothelial leakage, increased vascular permeability, and loss of vascular integrity.^[6,7]^ Therefore, the index was designed to integrate complementary biological pathways involved in the pathogenesis of pre-eclampsia into a single composite marker.

Importantly, the ELES index was developed based on predefined pathophysiological hypotheses rather than data-driven variable selection or statistical optimization procedures. RDW, AST, and albumin were selected a priori according to their established biological relevance to endothelial dysfunction, cellular injury, and erythrocyte stress in pre-eclampsia. Therefore, the index was intended as a biologically informed composite marker rather than an empirically optimized prediction model.

### 2.7. Statistical analysis

The data obtained in the study were analyzed statistically using SPSS vn. 27 software (Statistical Package for the Social Sciences). Descriptive statistics were reported as mean ± standard deviation (SD), median, minimum and maximum values for continuous variables and as number (n) and percentage (%) for categorical variables. Conformity of the data to normal distribution was assessed using the Shapiro–Wilk test and boxplot graphs.

In the comparison of 2 groups of continuous data showing normal distribution, Student *t* test was used. Relationships between variables were examined using Pearson or Spearman correlation analysis, depending on distribution. Pearson Chi-square test was applied in the comparisons of categorical data. Diagnostic performance analyses and ROC curve analysis were used to evaluate the power of the ELES index in predicting the presence of disease. To determine risk factors affecting pre-eclampsia, logistic regression analysis was performed. Results were evaluated in a 95% confidence interval, and the level of statistical significance was set at *P* < .05. The optimal cutoff value for the ELES index was determined using the Youden index derived from ROC curve analysis. Only participants with complete first-trimester laboratory measurements and complete delivery records were included in the final analysis; therefore, no missing data were present among the analyzed variables. No formal sensitivity analyses were performed. To avoid multicollinearity, the ELES index and its individual components (RDW, AST, and albumin) were not included in the same multivariable regression model.

## 3. Results

The hospital database was retrospectively screened for women who delivered at Kocaeli City Hospital during the preceding 3 years. Eligible participants were identified by cross-referencing the labor-ward delivery register with the laboratory information system to retrieve first-trimester biochemical results. Women who met all inclusion criteria and had complete first-trimester laboratory and delivery records were enrolled consecutively. Those with any exclusion criterion (multiple pregnancy, chronic hypertension, diabetes mellitus, liver disease, renal disease, hematological disease, active infection, or incomplete records) were removed. The final cohort comprised 118 women (38 pre-eclampsia cases, 80 normotensive controls). No missing data were present for the analyzed variables.

118 women were included in the final analysis, including 38 women who subsequently developed pre-eclampsia and 80 normotensive controls. All the included cases were aged a mean of 30.36 ± 5.17 years, ranging from 19 to 44 years. Mean gravida was determined to be 2.18 ± 1.04 and mean parity 1.18 ± 1.04. The gestational age at birth was a mean of 37.23 ± 2.91 weeks, and birthweight was a mean of 3056.86 ± 780.14 grams. The births were recorded as 35.6% normal spontaneous delivery and 64.4% cesarean delivery (Table 1).

**Table 1:** Distribution of descriptive characteristics.

		n (%)
Age (yr)	Mean ± SD	30.36 ± 5.17
	Median (min–max)	31 (19–44)
Gravida	1 gravida	37 (31.4)
	2 gravida	39 (33.1)
	≥3 gravida	42 (35.6)
	Mean ± SD	2.18 ± 1.04
	Median (min–max)	2 (1–5)
Parity	None	37 (31.4)
	1	39 (33.1)
	≥2	42 (35.6)
	Mean ± SD	1.18 ± 1.04
	Median (min–max)	1 (0–4)
Gestational age at birth (wk)	Mean ± SD	37.23 ± 2.91
	Median (min–max)	38 (28–40)
Birthweight	Mean ± SD	3056.86 ± 780.14
	Median (min–max)	3200 (600–4360)
Mode of delivery	NSD	42 (35.6)
	C/S	76 (64.4)
Group	Patient	38 (32.2)
	Control	80 (67.8)

NSD = normal spontaneous delivery, SD = standard deviation.

The characteristics of the laboratory findings of all the cases are shown in Table 2. The mean values determined were RDW: 13.59 ± 1.57, neutrophil count: 6.07 ± 1.77, lymphocyte count: 2.12 ± 0.63, thrombocyte count: 270.81 ± 75.22, MPV: 10.51 ± 0.94, AST: 16.58 ± 5.29, ALT: 15.64 ± 9.74, and albumin: 42.58 ± 3.24 (Table 2).

**Table 2:** Distribution of laboratory findings.

	Mean ± SD	Median (min–max)
RDW (%)	13.59 ± 1.57	13.3 (11–21)
NEU (×10^3^/μL)	6.07 ± 1.77	5.9 (1.7–10)
LY (×10^3^/μL)	2.12 ± 0.63	2.1 (0.7–4)
PLT (×10^3^/μL)	270.81 ± 75.22	263 (94–551)
MPV (fL)	10.51 ± 0.94	10.5 (8.8–13)
AST (U/L)	16.58 ± 5.29	15 (6–41)
ALT (U/L)	15.64 ± 9.74	13 (3–60)
Albumin (g/L)	42.58 ± 3.24	43 (33–51)

ALT = alanine aminotransferase, AST = aspartate aminotransferase, LY = lymphocyte count, MPV = mean platelet volume, NEU = neutrophil count, PLR = platelet-lymphocyte ratio, PLT = platelet count, RDW = red cell distribution width, SD = standard deviation.

According to the distribution of the systemic inflammation indexes of the cases, the mean values were determined to be ELES index: 5.27 ± 2.00, NLR:3.06 ± 1.35, PLR:138.66 ± 49.84 (Table 3).

**Table 3:** Distribution of systemic inflammation indexes.

	Mean ± SD	Median (min–max)
ELES index	5.27 ± 2.00	4.9 (1.8–16.1)
NLR	3.06 ± 1.35	2.8 (1–11)
PLR	138.66 ± 49.84	131.3 (40.8–402.5)

ELES = Endothelial Leakage–Erythrocyte Stress, NLR = neutrophil-lymphocyte ratio, PLR = platelet-lymphocyte ratio, SD = standard deviation.

In the comparisons of the clinical characteristics, no statistically significant difference was determined between the pre-eclampsia and control groups in respect of age, gravida, and parity (*P* > .05). The gestational age at birth and birthweight values were observed to be significantly lower in the patient group than in the control group (*P* = .001 for both). The rate of cesarean section deliveries was significantly higher in the patient group (*P* = .001; Table 4).

**Table 4:** Comparisons of the descriptive characteristics of the groups.

		Patient (n = 38)	Control (n = 80)	*P*
Age (yr)	Mean ± SD	30.89 ± 4.87	30.10 ± 5.32	.438†
	Median (min–max)	31.5 (23–44)	30 (19–44)	
Gravida	1 gravida	16 (42.1)	21 (26.3)	.213‡
	2 gravida	10 (26.3)	29 (36.3)	
	≥3 gravida	12 (31.6)	30 (37.5)	
	Mean ± SD	2.03 ± 1.13	2.25 ± 1.00	
	Median (min–max)	2 (1–5)	2 (1–4)	
Parity	None	16 (42.1)	21 (26.3)	.213‡
	1	10 (26.3)	29 (36.3)	
	≥2	12 (31.6)	30 (37.5)	
	Mean ± SD	1.03 ± 1.13	1.25 ± 1.00	
	Median (min–max)	1 (0–4)	1 (0–3)	
Gestational age at birth (wk)	Mean ± SD	34.32 ± 3.48	38.61 ± 0.93	.001†,**
	Median (min–max)	35 (28–40)	39 (36–40)	
Birthweight (gr)	Mean ± SD	2392.11 ± 963.14	3372.63 ± 391.62	.001†,**
	Median (min–max)	2397.5 (600–4360)	3355 (2570–4300)	
Manner of birth	NSD	3 (7.9)	39 (48.8)	.001‡,**
	C/S	35 (92.1)	41 (51.3)	

NSD = normal spontaneous delivery, SD = standard deviation.

† Student *t* test.

‡ Pearson chi square test.

** *P* < .01.

In the comparisons of the laboratory parameters and inflammation indexes, no significant difference was determined between the groups in respect of RDW, neutrophils, lymphocytes, thrombocytes, MPV, and ALT values (*P* > .05). In the patient group, the AST levels were found to be significantly higher (*P* = .013), and the albumin levels were significantly lower (*P* = .001; Table 5).

**Table 5:** Comparisons of the laboratory findings of the groups.

		Patient (n = 38)	Control (n = 80)	*P* †
RDW (%)	Mean ± SD	13.89 ± 1.50	13.45 ± 1.59	.148
	Median (min–max)	13.7 (11.9–18.6)	13 (11–21)	
NEU (×10^3^/μL)	Mean ± SD	6.29 ± 2.03	5.97 ± 1.63	.353
	Median (min–max)	5.9 (2.8–10)	6 (1.7–8.9)	
LY (×10^3^/μL)	Mean ± SD	2.14 ± 0.63	2.11 ± 0.64	.784
	Median (min–max)	2.1 (1–3.9)	2.1 (0.7–4)	
PLT (×10^3^/μL)	Mean ± SD	288.71 ± 96.84	262.31 ± 61.33	.130
	Median (min–max)	272.5 (94–551)	260.5 (115–415)	
MPV (fL)	Mean ± SD	10.47 ± 0.83	10.53 ± 0.99	.752
	Median (min–max)	10.3 (8.8–12)	10.6 (8.8–13)	
AST (U/L)	Mean ± SD	18.68 ± 6.88	15.59 ± 4.02	.013*
	Median (min–max)	17 (10–41)	15 (6–31)	
ALT (U/L)	Mean ± SD	16.71 ± 10.00	15.13 ± 9.63	.411
	Median (min–max)	15 (7–48)	12 (3–60)	
Albumin (g/L)	Mean ± SD	40.26 ± 3.41	43.68 ± 2.51	.001**
	Median (min–max)	40 (33–46)	44 (36.8–51)	

ALT = alanine aminotransferase, AST = aspartate aminotransferase, LY = lymphocyte count, MPV = mean platelet volume, NEU = neutrophil count, PLR = platelet-lymphocyte ratio, PLT = platelet count, RDW = red cell distribution width, SD = standard deviation.

† Student *t* test.

* *P *< .05.

** *P* < .01.

The ELES index values were seen to be significantly higher in the patient group than in the control group (6.36 ± 2.59 vs 4.75 ± 1.38, *P* = .001). No statistically significant difference was determined between the groups in respect of the NLR and PLR values (*P* > .05; Table 6).

**Table 6:** Comparisons of the systemic inflammation index values of the groups.

		Patient (n = 38)	Control (n = 80)	*P*
ELES index	Mean ± SD	6.36 ± 2.59	4.75 ± 1.38	.001**
	Median (min–max)	5.7 (3.2–16.1)	4.7 (1.8–10)	
NLR	Mean ± SD	3.10 ± 1.31	3.04 ± 1.38	.815
	Median (min–max)	2.9 (1–6.7)	2.8 (1.3–11)	
PLR	Mean ± SD	142.26 ± 51.04	136.95 ± 49.50	.590
	Median (min–max)	134.8 (40.8–263)	128.3 (63.2–402.5)	

ELES = Endothelial Leakage–Erythrocyte Stress, NLR = neutrophil-lymphocyte ratio, PLR = platelet-lymphocyte ratio, SD = standard deviation.

† Student *t* test.

** *P* < .01.

**Figure 1.**
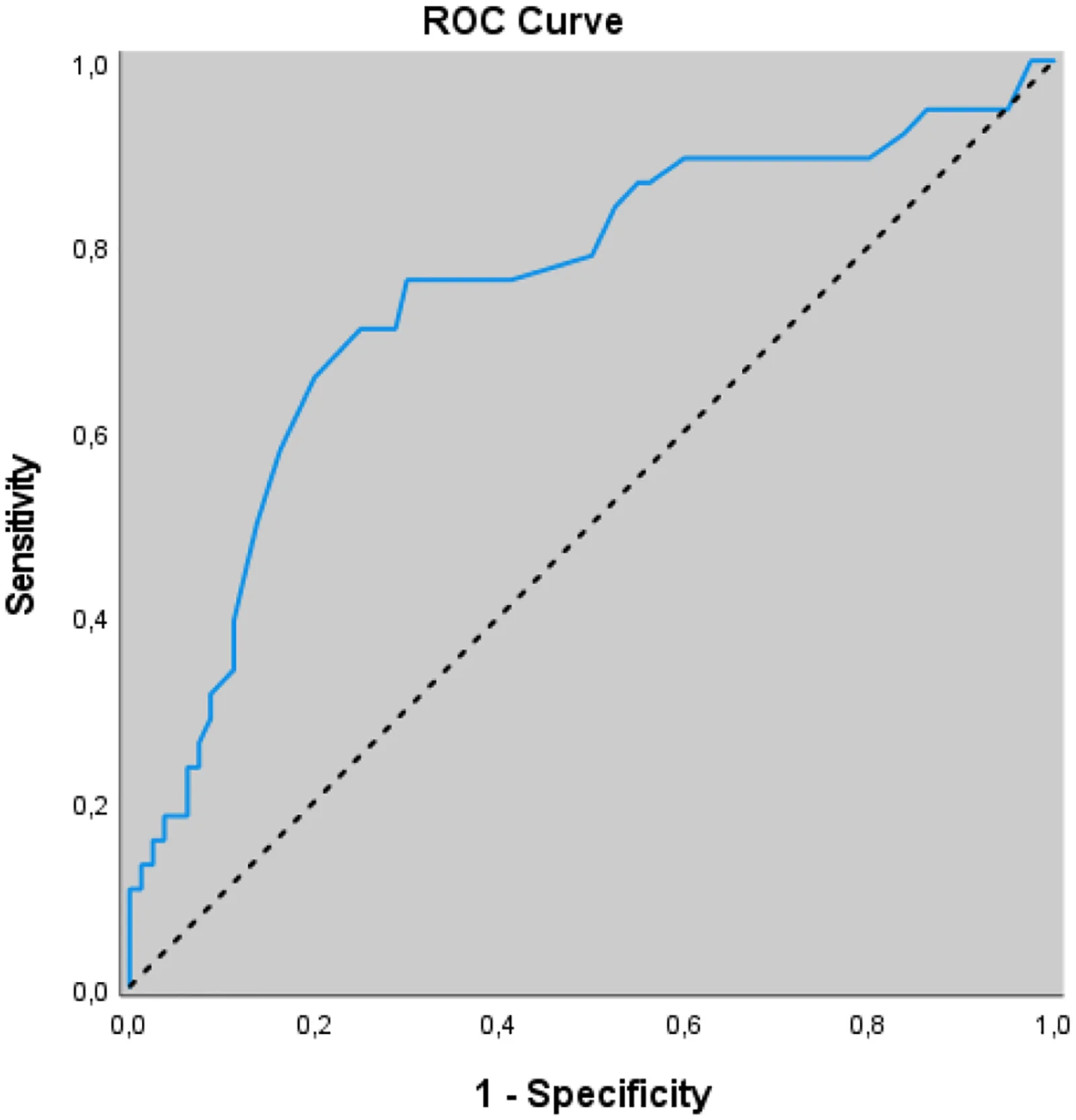
Receiver operating characteristic (ROC) curve of the Endothelial Leakage–Erythrocyte Stress (ELES) index for the prediction of pre-eclampsia. The area under the curve (AUC) was 0.75 (95% CI: 0.65–0.85). AUC = area under the curve, CI = confidence interval, ELES = Endothelial Leakage–Erythrocyte Stress, ROC = receiver operating characteristic.

The diagnostic performance results and ROC curve analysis results of the ELES index in the prediction of pre-eclampsia were examined. A cutoff value of 5 in the current series was determined to have a sensitivity of 76.3%, specificity of 65.0%, positive predictive value of 50.9%, and negative predictive value of 85.2%. ROC curve analysis demonstrated an area under the curve (AUC) value of 0.75 (95% CI: 0.65–0.85, *P* = .001), indicating moderate discriminatory performance for the prediction of pre-eclampsia. A statistically significant correlation was determined between the ELES index and the cutoff value of 5 for disease prediction (*P* = .001, *P* < .01). Women with an ELES index value ≥ 5 had 5.97-fold higher odds of developing pre-eclampsia (OR 5.971, 95% CI: 2.427–14.688, *P* = .001; Table 7 and Fig. 1).

**Table 7:** Results of the diagnostic scan tests and ROC curve analysis of the ELES index in the prediction of pre-eclampsia.

	Diagnostic scan					ROC curve		*P*
	Cut off	Sensitivity	Specificity	Positive predictive value	Negative predictive value	Area	95% confidence interval	
ELES index	*≥5*	76.32	65.0	50.9	85.2	0.750	0.650–0.850	.001**

ELES = Endothelial Leakage–Erythrocyte Stress, ROC = receiver operating characteristic.

** *P* < .01.

To further evaluate the discriminatory performance of the ELES index, ROC curves of the ELES index and its individual components (AST, albumin, and RDW) were compared using the DeLong test. The ELES index demonstrated an AUC of 0.750 (95% CI: 0.662–0.825), which was significantly higher than that of AST (AUC: 0.653, 95% CI: 0.560–0.739; *P* = .017) and RDW (AUC: 0.593, 95% CI: 0.499–0.683; *P* = .012). Albumin yielded the highest AUC value (0.775, 95% CI: 0.689–0.847); however, its discriminatory performance was not significantly different from that of the ELES index (*P* = .698). In addition, albumin demonstrated significantly better predictive performance than RDW (*P* = .011). No statistically significant differences were observed between AST and albumin (*P* = .113) or between AST and RDW (*P* = .427; Table 8).

**Table 8:** Comparison of ROC curves for the ELES Index, AST, albumin, and RDW in predicting disease presence.

Variable	AUC (95% CI)	Comparison	*P*-value (DeLong test)
ELES Index	0.750 (0.662–0.825)	ELES Index vs AST	.017*
AST(U/L)	0.653 (0.560–0.739)	ELES Index vs albumin	.698
Albumin(g/L)	0.775 (0.689–0.847)	ELES Index vs RDW	.012*
RDW(%)	0.593 (0.499–0.683)	AST vs Albumin AST vs RDW Albumin vs RDW	.113 .427 .011*

DeLong test was used to compare the areas under the ROC curves (AUCs).

AST = aspartate aminotransferase, AUC = area under the curve, CI = confidence interval, ELES = Endothelial Leakage–Erythrocyte Stress, RDW = red cell distribution width, ROC = receiver operating characteristic.

* Statistically significant differences are indicated by an asterisk (*P* < .05).

To identify independent predictors of pre-eclampsia, 2 separate multivariable logistic regression models were constructed to avoid multicollinearity between the ELES index and its individual components.

In the first multivariable logistic regression model, maternal age, gravida, and ELES index (≥5) were included. The analysis demonstrated that an ELES index value ≥ 5 was independently associated with the development of pre-eclampsia (OR: 5.971, 95% CI: 2.427–14.688, *P* = .001), whereas maternal age and gravida were not significantly associated with disease development (Table 9).

**Table 9:** Multivariable logistic regression analysis of risk factors associated with pre-eclampsia.

Variables	*B*	*P*	OR	95% CI lower	95% CI upper
Age	0.064	.156	1.066	0.976	1.164
Gravida (1)	–	.311	–	–	–
Gravida (2)	0.695	.205	2.005	0.683	5.882
Gravida (≥3)	−0.053	.923	0.948	0.321	2.802
ELES index (≥5)	1.787	.001**	5.971	2.427	14.688

CI = confidence interval, ELES = Endothelial Leakage–Erythrocyte Stress, OR = odds ratio.

** *P* < .01.

In the second multivariable logistic regression model, maternal age, gravida, RDW, AST, and albumin levels were evaluated. AST and albumin levels remained independently associated with pre-eclampsia. According to the model, each 1-unit increase in AST level was associated with a 1.125-fold increase in the odds of pre-eclampsia (OR: 1.125, 95% CI: 1.013–1.248, *P* = .028), whereas each 1-unit increase in albumin level was associated with a reduced risk of pre-eclampsia (OR: 0.651, 95% CI: 0.541–0.784, *P* = .001). RDW, maternal age, and gravida were not independently associated with pre-eclampsia in the adjusted model (Table 10).

**Table 10:** Multivariable logistic regression analysis of individual laboratory parameters associated with pre-eclampsia.

Variables	*B*	*P*	OR	95% CI lower	95% CI upper
Age	−0.008	.871	0.992	0.897	1.097
Gravida (1)	–	.515	–	–	–
Gravida (2)	0.710	.273	2.034	0.572	7.233
Gravida (≥3)	0.172	.783	1.188	0.349	4.046
RDW (%)	0.198	.197	1.219	0.902	1.646
AST (U/L)	0.117	.028*	1.125	1.013	1.248
Albumin (g/L)	−0.429	.001**	0.651	0.541	0.784

AST = aspartate aminotransferase, CI = confidence interval, RDW = red cell distribution width.

* *P* < .05.

** *P* < .01.

Overall, the findings of the present study demonstrated that the first-trimester ELES index was significantly higher in women who subsequently developed pre-eclampsia. ROC curve analysis showed that the ELES index had moderate discriminatory performance for predicting pre-eclampsia, with acceptable sensitivity and specificity at a cutoff value of≥5. In multivariable logistic regression analysis adjusted for maternal age and obstetric characteristics, the ELES index remained independently associated with the development of pre-eclampsia. In the component-based regression model, higher AST and lower albumin levels were also independently associated with disease development. These findings suggest that the ELES index may serve as a practical and low-cost biomarker reflecting endothelial dysfunction and erythrocyte stress in the early prediction of pre-eclampsia.

## 4. Discussion

The results obtained in this study showed that the ELES index, a new composite index derived from routine laboratory parameters in the first trimester, is associated with the development of pre-eclampsia. The finding that the ELES index was significantly higher in the pre-eclampsia group and that it showed differentiating power at a moderate level (AUC: 0.75) in the ROC analysis suggests that this index could be helpful in risk stratification in the early pregnancy period. In addition, the results of the multivariate logistic regression analysis showed that an ELES index value ≥ 5 was independently associated with the development of pre-eclampsia (OR 5.971, 95% CI: 2.427–14.68), thereby supporting the predictive value of the index. To the best of our knowledge, this study is one of the first to have investigated the role of a composite index evaluating endothelial leakage and erythrocyte stress together in the early prediction of pre-eclampsia. Unlike many prediction models derived through extensive variable screening and statistical optimization, the ELES index was constructed according to biological plausibility and established pathophysiological mechanisms. This conceptual approach may reduce susceptibility to model instability and facilitate reproducibility across different clinical settings.

Pre-eclampsia is a complex and multifactorial disease that develops as a result of abnormal placentation in which systemic endothelial dysfunction, inflammation, and microvascular damage together play a role.^[1-3]^ Placental hypoxia developing as a result of insufficient trophoblast invasion and disrupted spiral artery remodeling causes the release of anti-angiogenic factors (increased sFlt-1 and decreased PIGF) into the maternal circulation, leading to widespread endothelial damage and increased vascular permeability.^[4,5]^ This process creates a clinical spectrum of disease by combining oxidative stress and systemic inflammation.^[8]^ Studies in recent years have shown that pre-eclampsia is not only a placental disease but also a marker of maternal vascular response initiated by subclinical endothelial activation in the early period.^[15,16]^

Increased vascular permeability due to endothelial damage causes intravascular protein loss and a decrease in serum albumin levels. The current study results showed a significant decrease in albumin levels in the pre-eclampsia group. It has been previously reported in the literature that hypoalbuminemia is not only a hepatic synthesis disorder but is also a reflection of capillary leakage and oxidative stress.^[6,7]^ This supports that the inverse contribution of albumin in the ELES index is pathophysiologically significant. It has also been shown that low albumin levels are associated with increased inflammatory burden and endothelial dysfunction, and could be a reflection of increased vascular permeability.^[17,18]^

Microvascular damage and oxidative stress in pre-eclampsia lead to reduced deformability and an increase in erythrocyte size heterogeneity by disrupting erythrocyte membrane integrity. This is indirectly reflected as an increase in erythrocyte distribution width (RDW).^[9]^ Although a relationship of RDW with pre-eclampsia has been shown, the majority of studies in the literature have focused on the later weeks of pregnancy.^[10]^ The evaluation of RDW together with other parameters in the early period of pregnancy suggests that the predictive value of RDW, which is limited when used alone, could be increased with a composite index. It has also been shown in recent years that RDW is an independent marker of oxidative stress and microvascular dysfunction in cardiovascular diseases and systemic inflammatory conditions.^[19]^

AST, which is another component of the ELES index, may reflect hepatic cellular damage and endothelial-origin subclinical organ dysfunction. In pre-eclampsia, an increase has been observed in AST levels due to hepatic sinusoidal endothelial damage and microvascular ischemia. Therefore, the inclusion of AST in a composite structure presents a more holistic approach reflecting not only liver damage but also systemic endothelial dysfunction. Similarly, it has been reported that subclinical liver enzyme elevation could be an early marker of systemic endothelial dysfunction, associated particularly with the severity of pre-eclampsia.^[11]^

In the prediction of pre-eclampsia, various inflammatory and hematological indexes have been researched in recent years. Markers such as the NLR, PLR, and SII reflect systemic inflammation and have been found to be associated with pre-eclampsia. However, these parameters basically represent only the inflammatory component of the disease and do not sufficiently cover fundamental pathophysiological processes such as endothelial dysfunction and microvascular damage. It has also been reported in most studies that these indexes have been evaluated in the later weeks of pregnancy when the disease is clinically evident and remain limited in early prediction.^[9,10]^ A recent meta-analysis emphasized that these indexes have heterogeneous performance and are insufficient alone for clinical decision-making.^[20]^

In this context, the findings obtained in the current study are noteworthy when compared with the inflammation-based hematological indexes frequently used in the literature. While no significant difference was determined between the pre-eclampsia and control groups in respect of the NLR and PLR, the ELES index was seen to have higher differentiating power in the prediction of pre-eclampsia. This suggests that inflammation alone, although an important component in the pathogenesis of pre-eclampsia, may not provide a sufficient prediction of the disease in the early period. The fact that the ELES index showed higher predictive performance may be attributed to this index evaluating not only inflammation but also endothelial dysfunction (albumin), cellular damage (AST), and erythrocyte stress (RDW) together. This multidimensional approach allows a more comprehensive reflection of the early pathophysiology of pre-eclampsia.

The Endothelial Activation and Stress Index (EASIX), one of the composite indexes evaluating endothelial dysfunction, is defined as a marker reflecting microvascular damage.^[13]^ However, EASIX is usually used for prognostic purposes in hematological malignancies and critical diseases, and its use in the field of obstetrics is extremely new. Recent studies have shown that EASIX is associated with adverse maternal and perinatal outcomes in pre-eclampsia cases, but data related to predictive use, especially in the early period of pregnancy, have been reported to still be limited.^[21,22]^

Although it includes parameters such as lactate dehydrogenase, creatinine, and thrombocyte count, EASIX does not directly reflect erythrocyte morphology. This suggests that the inclusion of the RDW component in the ELES index could theoretically have more extensive pathophysiological coverage. In this context, by reflecting endothelial dysfunction (albumin), cellular damage (AST), and erythrocyte stress (RDW) together, unlike other available indexes, the ELES index may provide a more holistic evaluation of the multidimensional pathophysiology of pre-eclampsia. In contrast to single biomarkers, the use of these 3 parameters together provides a comprehensive risk evaluation by integrating hematological changes due to both vascular permeability and microvascular damage.

Notably, although albumin alone yielded the highest AUC value among the individual components (0.775, 95% CI: 0.689–0.847), its discriminatory performance did not differ significantly from that of the ELES index (DeLong *P* = .698), whereas the ELES index significantly outperformed both AST (AUC: 0.653, 95% CI: 0.560–0.739; *P* = .017) and RDW (AUC: 0.593, 95% CI: 0.499–0.683; *P* = .012) individually. Since albumin constitutes one of the 3 components of the composite index, statistical equivalence between the ELES index and albumin alone does not, in itself, demonstrate that the composite structure confers discriminatory value beyond that of albumin. Rather, the apparent benefit of the composite approach appears to derive primarily from its superiority over the weaker individual components (AST and RDW) rather than from surpassing the strongest one. Nonetheless, the composite structure may still offer conceptual value by integrating 3 distinct pathophysiological dimensions of pre-eclampsia: endothelial dysfunction (albumin), hepatocellular and endothelial injury (AST), and erythrocyte stress (RDW), within a single, biologically interpretable index derived from routinely available first-trimester parameters.

Compared with the Fetal Medicine Foundation (FMF) first-trimester screening algorithm, which incorporates maternal characteristics, mean arterial pressure, uterine artery Doppler findings, and biochemical markers such as PlGF and PAPP-A, the ELES index represents a simpler and more accessible alternative based solely on routinely available laboratory parameters.^[23,24]^ While FMF-based models offer high predictive accuracy for early-onset pre-eclampsia, their implementation requires specialized equipment and biomarkers, which may limit their applicability in resource-constrained settings. In contrast, the ELES index may serve as a low-cost, widely accessible adjunctive screening tool for early risk stratification.

In addition, an ELES index value ≥ 5 was associated with approximately 6-fold higher odds of developing pre-eclampsia (OR 5.971, 95% CI: 2.427–14.688), suggesting a potentially clinically meaningful threshold for risk classification. Current guidelines emphasize the importance of combined biomarker approaches for early prediction of pre-eclampsia.^[25]^ In this context, the ELES index may be considered a low-cost and accessible biomarker derived entirely from routine laboratory parameters, offering practical utility for early risk assessment in clinical practice.

In a practical clinical scenario, the ELES index could be automatically calculated from routine first-trimester laboratory tests (RDW, AST, and albumin) obtained at 11–13 + 6 weeks of gestation. Women with an ELES index ≥ 5 may be identified as a higher-risk group and may benefit from closer antenatal surveillance, early placental evaluation, and consideration of preventive interventions such as low-dose aspirin when appropriate. This approach may facilitate early triage without additional cost or specialized testing, particularly in settings where advanced predictive tools are not readily available. Importantly, the ELES index should be considered an adjunct rather than a replacement for established screening models.

Nevertheless, the findings should be interpreted with caution because maternal body mass index (BMI) data were not available for analysis. Since obesity is a well-established risk factor for pre-eclampsia and may influence several laboratory parameters included in the ELES index, residual confounding cannot be excluded, and the independent contribution of obesity-related metabolic factors to the observed associations remains uncertain.

The main strength of this study is the evaluation of a novel, low-cost composite index derived from routine laboratory parameters and its assessment in the first trimester of pregnancy. However, several limitations should be acknowledged. The retrospective, single-center design and the relatively small sample size limit the robustness and generalizability of the findings.

Because the ELES index was developed and evaluated within the same study population, the possibility of optimism bias cannot be completely excluded. However, the index was not generated through empirical model optimization or multiple-variable screening strategies; rather, it was based on predefined biologically relevant parameters selected according to current knowledge of pre-eclampsia pathophysiology. This approach may enhance the generalizability of the index, although external validation remains essential.

The relatively small sample size was mainly attributable to the retrospective design and the fragmented nature of antenatal care in routine clinical practice. Pregnant women are not uniformly followed within a single healthcare facility throughout pregnancy, and some patients who underwent first-trimester assessment at our institution delivered elsewhere. Conversely, a subset of women presented only at delivery without prior antenatal follow-up data. As a result, complete longitudinal clinical and laboratory data were available for only a limited number of cases, which may have introduced selection bias. Furthermore, because the exact number of initially screened and excluded patients could not be reliably retrieved from the electronic database, a participant flow diagram could not be constructed.

Another limitation relates to the availability of first-trimester serum albumin measurements. Although serum albumin is not part of the standardized national antenatal screening protocol, it is frequently included in the biochemistry panel ordered at the first obstetric visit at our institution, at the discretion of the attending physician. As this ordering practice is not governed by a fixed institutional protocol, we cannot fully exclude the possibility that clinical judgment influenced which patients had albumin measured, introducing a potential selection bias. However, since the study population consisted of women already presenting for routine first-trimester follow-up, rather than a subgroup selectively referred for suspected preeclampsia, we consider this risk to be limited. All eligible patients meeting the inclusion criteria and lacking any predefined exclusion criteria were included, regardless of outcome.

As the study was conducted as a retrospective case-control analysis with an enriched pre-eclampsia cohort, the reported predictive performance measures, including sensitivity and specificity, may not accurately reflect performance in an unselected general obstetric population. In addition, maternal BMI data were not consistently available in the hospital records because anthropometric measurements were recorded in different outpatient systems that were not fully integrated with the delivery database. Therefore, BMI information could not be reliably retrieved for a substantial proportion of patients, precluding adjustment for BMI and potentially introducing residual confounding related to obesity and obesity-associated metabolic factors.

Information regarding low-dose aspirin prophylaxis was not consistently documented in the retrospective records. Therefore, the potential influence of aspirin use on the observed associations could not be evaluated.

Furthermore, detailed information regarding severe features and gestational age at diagnosis of pre-eclampsia was not consistently available in the retrospective records. Therefore, analyses according to disease severity and classification into early-onset and late-onset pre-eclampsia could not be performed.

## 5. Conclusion

The present study demonstrated that first-trimester ELES index values were significantly associated with the subsequent development of pre-eclampsia and remained independently associated with disease risk after multivariable adjustment. These findings should be considered exploratory and hypothesis-generating. Although the ELES index showed promising potential as a practical and low-cost biomarker for early risk stratification, it should currently be regarded as a complementary risk stratification tool rather than a stand-alone predictive test. Prospective multicenter studies with larger, independent, and unselected obstetric populations, together with external validation and decision-curve analysis, are required to confirm its clinical utility before routine implementation can be recommended.

## Author contributions

**Formal analysis:** Funda Demirel.

**Methodology:** Funda Demirel.

**Project administration:** Funda Demirel.

**Supervision:** Berrin Oztas.

**Visualization:** Berrin Oztas.

**Writing – original draft:** Funda Demirel, Berrin Oztas.

**Writing – review & editing:** Funda Demirel, Berrin Oztas.
